# Type I Interferon Signaling Is Required for Dacryoadenitis in the Nonobese Diabetic Mouse Model of Sjögren Syndrome

**DOI:** 10.3390/ijms19103259

**Published:** 2018-10-20

**Authors:** Yury Chaly, Jennifer Y. Barr, David A. Sullivan, Helen E. Thomas, Thomas C. Brodnicki, Scott M. Lieberman

**Affiliations:** 1 Stead Family Department of Pediatrics, Carver College of Medicine, University of Iowa, Iowa City, IA 52242, USA; yvchaly@gmail.com (Y.C.); jennifer-barr@uiowa.edu (J.Y.B.); 2 Department of Ophthalmology, Schepens Eye Research Institute of Massachusetts Eye and Ear, Harvard Medical School, Boston, MA 02114, USA; david.sullivan@schepens.harvard.edu; 3 Department of Medicine, St. Vincent’s Hospital, St. Vincent’s Institute, The University of Melbourne, Fitzroy, Victoria 3065, Australia; hthomas@svi.edu.au (H.E.T.); tbrodnicki@svi.edu.au (T.C.B.); 4 Interdisciplinary Graduate Program in Immunology, University of Iowa, Iowa City, IA 52242, USA

**Keywords:** Sjögren syndrome, lacrimal glands, nonobese diabetic mice, regulatory T cells, type I interferon, chemokines

## Abstract

Nonobese diabetic (NOD) mice spontaneously develop lacrimal and salivary gland autoimmunity similar to human Sjögren syndrome. In both humans and NOD mice, the early immune response that drives T-cell infiltration into lacrimal and salivary glands is poorly understood. In NOD mice, lacrimal gland autoimmunity spontaneously occurs only in males with testosterone playing a role in promoting lacrimal gland inflammation, while female lacrimal glands are protected by regulatory T cells (Tregs). The mechanisms of this male-specific lacrimal gland autoimmunity are not known. Here, we studied the effects of Treg depletion in hormone-manipulated NOD mice and lacrimal gland gene expression to determine early signals required for lacrimal gland inflammation. While Treg-depletion was not sufficient to drive dacryoadenitis in castrated male NOD mice, chemokines (*Cxcl9*, *Ccl19*) and other potentially disease-relevant genes (*Epsti1*, *Ubd*) were upregulated in male lacrimal glands. Expression of *Cxcl9* and *Ccl19*, in particular, remained significantly upregulated in the lacrimal glands of lymphocyte-deficient NOD-severe combined immunodeficiency (SCID) mice and their expression was modulated by type I interferon signaling. Notably, *Ifnar1*-deficient NOD mice did not develop dacryoadenitis. Together these data identify disease-relevant genes upregulated in the context of male-specific dacryoadenitis and demonstrate a requisite role for type I interferon signaling in lacrimal gland autoimmunity in NOD mice.

## 1. Introduction

Sjögren syndrome is a complex autoimmune disease characterized by lymphocytic infiltration of lacrimal and salivary glands leading to gland dysfunction and downstream complications including profound dry eyes and dry mouth, potentially vision-threatening ocular surface damage and poor oral health. Currently, the specific causes of Sjögren syndrome are not known and treatments to halt the autoimmune process are lacking. Because the diagnosis of Sjögren syndrome occurs years (even decades) after the initiation of autoimmunity [1,2], the initial inflammatory mechanisms are not well studied in humans. Fortunately, several animal models have been described that develop lacrimal and/or salivary gland autoimmunity with similar features as those observed in humans with Sjögren syndrome and these models have provided insights into the immunological mechanisms of autoimmunity [3,4]. Murphy Roths Large/lymphoproliferation (MRL/lpr) mice develop lymphoproliferation and spontaneous autoimmunity affecting several organs, including salivary glands (males and females) and lacrimal glands (females) [4,5]. Nonobese diabetic (NOD) mice spontaneously develop a Sjögren syndrome-like autoimmune attack on lacrimal and salivary glands with features more closely modelling human Sjögren syndrome including characteristic histopathological lesions (i.e., focal sialadenitis and focal dacryoadenitis), some relevant autoantibodies and dysfunction of the exocrine glands [3,4]. Thus, NOD mice provide a model to study cellular and molecular pathways that drive the pathogenic T cells to overcome regulatory mechanisms and invade the affected glands.

While Sjögren syndrome in humans is typically associated with both lacrimal and salivary gland manifestations, NOD mice do not spontaneously develop autoimmunity of both lacrimal and salivary glands. Rather, male NOD mice spontaneously develop lacrimal gland autoimmunity, while female NOD mice spontaneously develop salivary gland autoimmunity [5,6,7,8,9,10]. Prior work by us and others has demonstrated that lacrimal gland disease in male NOD mice is dependent on male sex hormones [7,8,10]. We have also previously demonstrated that lacrimal gland disease in male NOD mice is associated with dysfunction of lacrimal gland-protective regulatory T cells (Tregs), whereas female NOD mice have Tregs preventing lacrimal gland autoimmunity [8]. This is directly associated with the presence or absence of testosterone, though whether testosterone drives lacrimal gland autoimmunity through a direct effect on Tregs, effector T cells, other immune cells, or lacrimal gland epithelial cells has not been formally evaluated.

The purpose of this study was to identify immunological pathways associated with testosterone-mediated lacrimal gland autoimmunity in NOD mice. We found that protection from lacrimal gland autoimmunity in castrated male NOD mice did not require Tregs, suggesting that testosterone affects other immune cells or lacrimal gland epithelial cells to drive lacrimal gland inflammation. Through gene expression studies driven by analyses of prior microarray data, we identified four genes upregulated in the context of male-specific lacrimal gland inflammation in NOD mice. Upregulation of three of these genes was dependent on type I interferon (IFN), which has been implicated in the development of Sjögren syndrome in mice and humans [11,12]. Moreover, we found that NOD mice with disrupted type I IFN signaling were protected from lacrimal gland inflammation. Together our findings implicate testosterone as a trigger for the type I IFN pathway that is required for lacrimal gland autoimmunity in NOD mice providing a model in which to further dissect the specific immunological molecular mechanisms required for T-cell infiltration into lacrimal glands in the context of lacrimal gland autoimmunity.

## 2. Results

### 2.1. Transient Treg Depletion Is Not Sufficient to Drive Dacryoadenitis in Castrated Male NOD Mice

**Figure 1:**
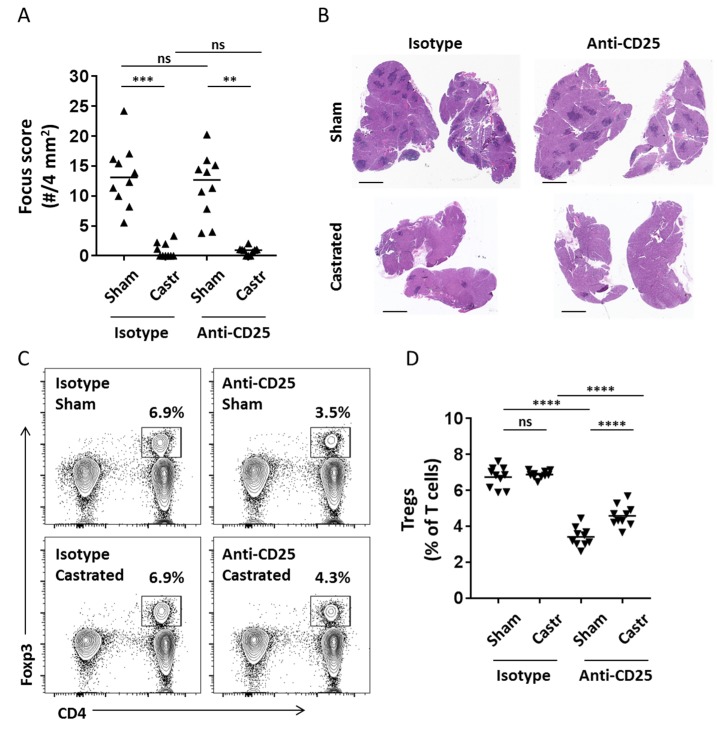
Treg-depletion is not sufficient to drive dacryoadenitis in castrated male NOD mice. (**A**) Quantitation of dacryoadenitis in castrated (Castr) or Sham-castrated (Sham) mice treated with Treg-depleting anti-CD25 antibody or isotype control (10 mice per group). Focus score equals # inflammatory foci per 4 mm^2^, where a focus requires a minimum of 50 mononuclear cells. Symbols represent individual mice, lines are medians. *p* < 0.0001 by Kruskal-Wallis with individual comparison *p* values by Dunn multiple comparisons test as shown: ** *p* < 0.01, ***p < 0.001, ^ns^ *p* > 0.05. (**B**) Representative hematoxylin and eosin stained sections of lacrimal glands from mice in **A**. Focus scores (# foci/4 mm^2^) for these samples are (clockwise from upper left): 10 (isotype/sham), 7.8 (anti-CD25/sham), 0.6 (anti-CD25/castrated), 2.2 (isotype/castrated). Scale bars = 1 mm. (**C**) Representative flow cytometry plots of cervical lymph node cells from mice in (**A**) with groups indicated on each plot. Plots are gated on T cells (CD3ε^+^ CD19^−^ singlets). Numbers are % cells within the indicated Treg gates. (**D**) Cumulative quantitation of Tregs (as % of T cells) in cervical lymph nodes of mice from (**A**,**B**). Symbols represent individual mice, lines are means. *p* < 0.0001 by two-tailed, one-way ANOVA with individual comparison *p* values by Holm-Sidak multiple comparisons test as shown: **** *p* < 0.0001; ^ns^ *p* > 0.05.

**Figure A1:**
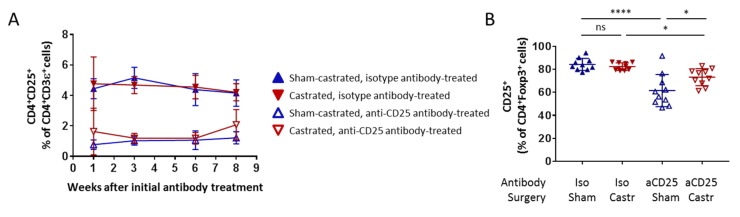
Treg depletion was comparable in castrated and sham-castrated mice. (**A**) Castrated (red) and sham-castrated (blue) mice in Figure 1 were treated with anti-CD25 monoclonal antibody (clone PC61; open symbols) to transiently deplete Tregs or isotype control antibody (closed symbols) as described in Figure 1. At 1, 3, 6, and 8 weeks following initial monoclonal antibody treatment, peripheral blood mononuclear cells were analyzed by flow cytometry to quantify the degree of Treg-depletion. Graph depicts CD4^+^CD25^+^ T cells as percentage of CD4 T cells (i.e., CD4^+^CD3e^+^) at time points as indicated. Symbols represent means, error bars are standard deviations. *n* = 10 mice per group. For these studies, a different clone of anti-CD25 monoclonal antibody (7D4) than that used for Treg-depletion was used for flow cytometry analyses. Repeated measures two-way ANOVA demonstrated significance based on mouse group (*p *< 0.0001) but not time (*p* = 0.6834) and with a significant interaction between the two (*p* = 0.0238). Values for the anti-CD25 antibody-treated groups were significantly decreased at each time point compared to the isotype-treated antibody groups (*p *< 0.0001 for each comparison) based on Tukey’s multiple comparisons test, but no differences were significant when comparisons were made between castrated and sham-castrated groups treated with the same antibody (i.e., anti-CD25-treated sham vs castrated OR isotype-treated sham vs castrated). Of note, the 8 week time point (2 weeks prior to take-down) suggested a trend towards an increase in Tregs in the castrated, anti-CD25-treated group compared to the sham-castrated anti-CD25 antibody-treated group; however, this was not statistically significant at this time point. (**B**) Graph depicts CD25^+^ cells as percentage of Tregs (i.e., CD4^+^Foxp3^+^) isolated from cervical lymph nodes of mice in Figure 1 and Figure A1A at take-down (10 weeks after initial antibody treatment). Symbols represent individual mice, lines are means, and error bars are standard deviations. *p* < 0.0001 by one-way ANOVA with individual comparisons corrected for multiple comparisons by Holm-Sidak as indicated: * *p* < 0.05, **** *p* < 0.0001, ^ns ^*p* > 0.05. Abbreviations: aCD25, anti-CD25; Iso, isotype; Castr, castrated; Sham, sham-castrated.

We recently reported that transient partial depletion of Tregs in vivo in female NOD mice using a Treg-depleting monoclonal antibody (anti-CD25 clone PC61) was sufficient to drive dacryoadenitis [6]. Castrated male NOD mice were also protected from development of dacryoadenitis [7,10]. To determine if castrated male NOD mice were protected from the development of dacryoadenitis due to the presence of a functional lacrimal gland-protective Treg population similar to female NOD mice, we treated castrated and sham-castrated male NOD mice with either the Treg-depleting anti-CD25 monoclonal antibody or with an isotype control antibody. Similar to previous studies [7,10], castration resulted in a marked decrease in the development of dacryoadenitis (Figure 1A,B, isotype group, sham vs. castrated). As shown in our prior study [6], treatment with an anti-CD25 monoclonal antibody did not alter development of dacryoadenitis in non-castrated male NOD mice (Figure 1A, isotype-sham vs. anti-CD25-sham). Surprisingly, treatment with the anti-CD25 monoclonal antibody failed to induce dacryoadenitis in castrated male NOD mice (Figure 1A,B) despite a significantly decreased Treg population in Treg-depleted mice (Figure 1C,D). While the castrated, Treg-depleted mice had a slight but significantly increased proportion of Tregs compared to the sham-castrated, Treg depleted mice at the end of the experiment (Figure 1D), their decreased proportion of Tregs compared to the non-Treg-depleted groups was comparable to the degree of Treg depletion we previously showed to be sufficient to induce dacryoadenitis in female NOD mice [6]. This difference in proportion of Tregs in the Treg-depleted groups at take-down of the experiment may be due to a decrease in the degree of Treg depletion in the castrated group or, alternatively, to a more rapid recovery of Tregs following the transient depletion. We favor the latter because measures of the proportions of CD4^+^CD25^+^ T cells within the peripheral blood mononuclear cells of mice treated with anti-CD25 monoclonal antibody were comparable between castrated and sham groups (Figure A1A). Thus, depletion of the CD25-expressing Tregs was similar between castrated and sham-castrated groups. Importantly, the proportion of Tregs expressing CD25 was comparable between isotype-treated castrated or sham-castrated groups at take-down suggesting there is no difference in CD25-expression on Tregs of castrated or sham-castrated mice. And, while CD25-expressing Tregs were lower in the Treg-depleted groups compared to isotype antibody-treated groups, the castrated mice exhibited a significantly higher proportion of CD25-expressing Tregs in cervical lymph nodes at take-down (Figure A1B). This suggested against an outgrowth of CD25^−^ Tregs in the castrated group, though association of testosterone with decreased CD25 expression on Tregs following Treg-depletion suggested that testosterone may have contributed to the slower recovery of Treg repopulation in the sham-castrated, Treg-depleted mice by antagonizing the upregulation of the high-affinity IL2 receptor on Treg populations. Together, these data demonstrate that Treg-depletion in castrated male NOD mice is not sufficient to drive dacryoadenitis in the same way that Treg-depletion drives dacryoadenitis in female NOD mice [6].

### 2.2. Identification and Validation of Disease-Relevant Genes Upregulated in Lacrimal Glands of Male NOD Mice

**Figure 2:**
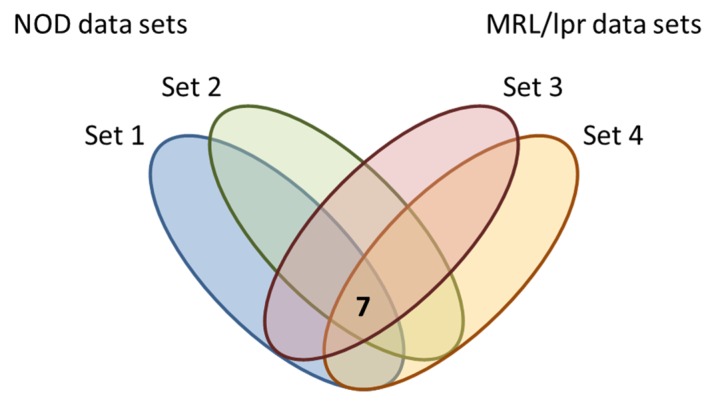
Identification of disease-relevant genes in lacrimal glands of mouse models of Sjögren syndrome. Microarray data sets described elsewhere were analyzed to identify disease-relevant genes upregulated in the context of lacrimal gland inflammation in four different scenarios based on sex or exposure to testosterone. The NOD mouse lacrimal gland data sets include genes upregulated at least 2-fold in male compared to female mice (set 1) or testosterone-treated female compared to placebo-treated female mice (set 2) as testosterone exposure is associated with dacryoadenitis in NOD mice. The MRL/lpr lacrimal gland data sets include genes upregulated at least 2-fold in female compared to male mice (set 3) or placebo-treated female compared to testosterone-treated female mice (set 4) as testosterone is relatively protective and associated with decreased dacryoadenitis in MRL/lpr mice. Each of the 4 data sets depicted in the figure represents the gene sets that were upregulated at least 2-fold for the indicated two-group comparison in analyses of two different microarray platforms (Affymetrix and CodeLink platforms–as noted in Table 1) for the indicated comparison. The intersection of all data sets resulted in 7 genes (see Table 1).

The lack of any increase in dacryoadenitis in the Treg-depleted, castrated mice in the studies above suggested that direct effects by testosterone on effector T cells or other non-Treg cells (e.g., innate immune cells, lacrimal gland epithelial cells) are required to drive the inflammatory response. Testosterone affects lacrimal gland size and morphology, as well as gene expression profiles [13,14,15,16,17]. Thus, the possibility remained that the dacryoadenitis-promoting effects of testosterone acted directly through modulation of the lacrimal gland environment. To address this, we analyzed microarray data sets (NCBI Gene Expression Omnibus GSE5876 and GSE5877) from experiments designed to identify sex- and testosterone-mediated gene expression changes in lacrimal glands in two mouse models of Sjögren syndrome: NOD mice and MRL/lpr mice. Briefly, NOD mice develop dacryoadenitis in a testosterone-dependent manner (e.g., males and testosterone-treated females), while female MRL/lpr mice develop dacryoadenitis and testosterone is protective (e.g., males and testosterone-treated females are protected) [10,18,19]. We compared data sets of genes upregulated at least 2-fold in the context of dacryoadenitis in each two-group comparison of microarray gene expression data to specifically identify genes that were upregulated in the context of dacryoadenitis in NOD and MRL/lpr mice—i.e., upregulated in male NOD mice compared to female NOD mice, upregulated in testosterone-treated female NOD mice compared to placebo-treated female NOD mice, upregulated in female MRL/lpr mice compared to male MRL/lpr mice, upregulated in placebo-treated female MRL/lpr mice compared to testosterone-treated female MRL/lpr mice. We reasoned that those genes upregulated in all of these four independent microarray analysis groups would be more likely to be disease-relevant given their upregulation in the context of dacryoadenitis in multiple different scenarios in different mouse strains. Only seven genes were significantly upregulated in the context of dacryoadenitis in all four independent microarray analyses (Figure 2, Table 1). In particular, we were interested in genes with potential roles in mechanisms upstream of lymphocyte infiltration into the lacrimal glands, which identified *Cxcl9*, *Ccl19*, *Epsti1*, *Ubd* and *Clec7a* as candidates for further study.

Using quantitative RT-PCR analyses of lacrimal glands from male and female NOD mice, we validated the upregulation of four of the five genes included in our study in lacrimal glands from male NOD mice compared to female NOD mice (Table 2). Moreover, to further support the association of upregulation of these disease-relevant genes with testosterone, we quantified gene expression in lacrimal glands of castrated and sham-castrated male NOD mice. Similar to upregulation in lacrimal glands of male compared to female NOD mice (Table 2), relative expression levels of *Cxcl9*, *Ubd*, *Epsti1* and *Ccl19* were increased in the lacrimal glands of sham-castrated compared to castrated male NOD mice (Table 3). In addition, relative gene expression of *Clec7a* was also increased in lacrimal glands of sham-castrated compared to castrated male NOD mice (Table 3). *Cxcl9*, *Ccl19* and *Epsti1* have been implicated in Sjögren syndrome in humans or animal models [20,21,22,23,24,25,26,27,28,29,30]. While *Ubd* has not, to our knowledge, been implicated in Sjögren syndrome, it may play a role in innate immunity and is therefore potentially relevant in early disease development. Thus, our analysis has identified *Cxcl9*, *Ccl19*, *Epsti1* and *Ubd* as genes whose expression may be modulated by testosterone in NOD lacrimal glands and may be contributing to the early stages of disease development.

### 2.3. Disease-Relevant Chemokine Genes Are Significantly Upregulated in Lymphocyte-Deficient NOD-SCID Mice

**Figure 3:**
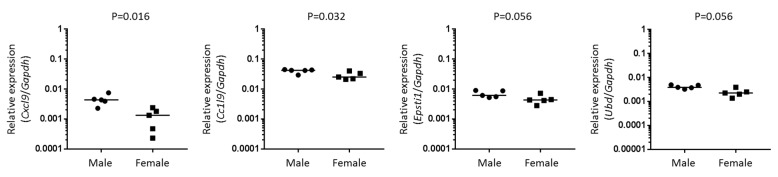
Disease-relevant chemokine genes are still significantly upregulated in male lacrimal glands from lymphocyte-deficient NOD-SCID mice. Graphs depict gene expression of indicated genes normalized to that of *Gapdh* in lacrimal glands of 14-16 week old male or female NOD-SCID mice. Symbols represent individual mice, lines are medians. *p* values by Mann-Whitney test applied to log-transformed ratios.

To determine if these disease-relevant genes were upregulated in the absence of lymphocytic infiltration, we analyzed gene expression in lacrimal glands of male and female NOD-SCID mice. The marked differences in levels of gene expression in male versus female NOD mice, with ratios of medians ranging 4.6–60 (Table 2), were no longer evident in the lacrimal glands from NOD-SCID mice (Figure 3). However, expression of *Cxcl9* and *Ccl19* each showed a slight but statistically significant, increase in expression in male compared to female NOD-SCID mice with ratios of medians of 3.3 and 1.7, respectively. While the biological relevance is not clear, these data suggest that these chemokine signals may begin to increase prior to T-cell infiltration.

### 2.4. Type I Interferon Signaling Affects Disease-Relevant Gene Expression and Is Required for Dacryoadenitis

**Figure 4:**
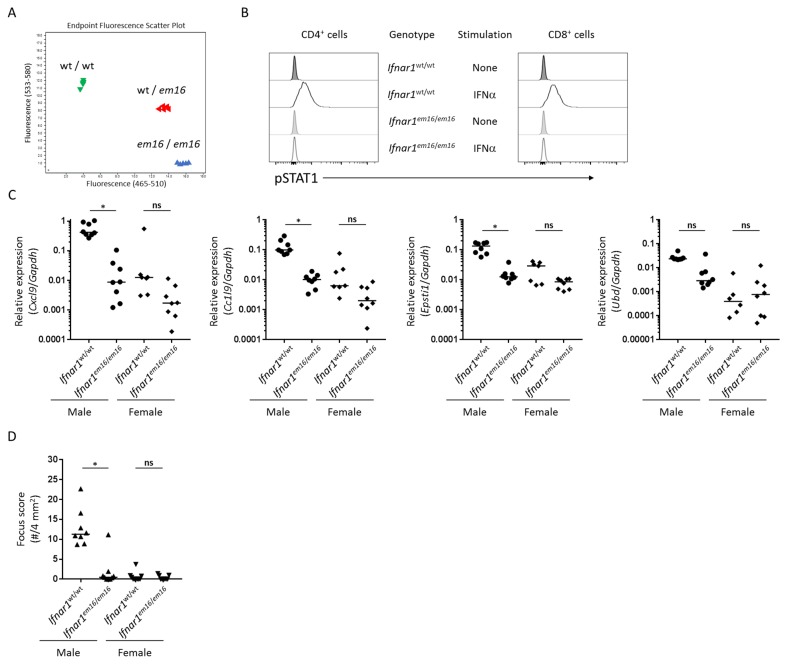
Type I IFN signaling is required for dacryoadenitis in male NOD mice. (**A**) Disruption of *Ifnar1* in NOD mice was confirmed by genotyping with Taqman probe assay for the *em16* mutant allele −5 bp deletion. (**B**) Functional deficiency in IFNAR1 signaling was confirmed by flow cytometry. Whole blood from wild-type (*Ifnar1*^wt/wt^) or *Ifnar1*-deficient (*Ifnar1^em16^*^/*em16*^) mice was stimulated with IFNα for 30 min then fixed and analyzed by flow cytometry for phosphorylation of STAT1 (pSTAT1). Histograms show pSTAT1 staining for CD4^+^ cells (left) or CD8^+^ cells (right). Cells not exposed to IFN stimulation were included as no stimulation controls. Similar levels of pSTAT1 staining was apparent from each group when stimulated with IFNγ as positive control (not shown). Data are representative of 2 mice per genotype. (**C**) Graphs depict gene expression of indicated genes normalized to that of *Gapdh* in lacrimal glands of 14–16 week old wild-type *(Ifnar1*^wt/wt^) or *Ifnar1*-deficient (*Ifnar1^em16^*^/*em16*^) male or female NOD mice as indicated on x-axis. Symbols represent individual mice, lines are medians. Relative expression ratios were log-transformed for statistical analysis. *p* < 0.001 for each gene data set by Kruskal-Wallis with *p* values for individual comparisons by Dunn multiple comparisons test as indicated for sex-matched wild-type versus *Ifnar1*-deficient mice: * *p* < 0.05, ^ns^ *p* > 0.05. Individual mouse data points for wild-type males and females are also summarized in Table 2. (**D**) Graph depicts focus scores (as defined in Figure 1) as quantitation of dacryoadenitis in lacrimal glands of 14–16 week old wild-type (*Ifnar1*^wt/wt^) or *Ifnar1*-deficient (*Ifnar1^em16^*^/*em16*^) male and female NOD mice. Symbols represent individual mice, lines are medians. *p* = 0.0003 by Kruskal-Wallis with individual comparisons by Dunn multiple comparisons test as indicated for sex-matched wild-type versus *Ifnar1*-deficient mice: * *p* = 0.018, ^ns^ *p* > 0.05.

One thing in common among these disease-relevant genes is their upregulation by interferons (IFN). Several of these genes are well-characterized as being upregulated by type II IFN but a search of the interferome database of interferon-regulated genes [31] identified that each is upregulated by type I IFN as well as type II IFN. Prior studies demonstrated that type II IFN (i.e., IFNγ) played a requisite role in salivary gland autoimmunity in NOD mice but the lack of type II IFN signaling (either in IFNγ-deficient or IFNγ receptor-deficient NOD mice) did not abrogate dacryoadenitis [32]. Type I IFN was shown to play a role in dacryoadenitis in a related mouse strain [11] but to our knowledge the role of type I IFN has not been evaluated in NOD mice. We generated NOD mice deficient in type I IFN signaling by CRISPR/cas9-mediated disruption of the *Ifnar1* gene (NOD.*Ifnar1^em16^*^/*em16*^, Figure 4A,B). Functional deficiency of IFNAR1 was confirmed by the lack of STAT1 phosphorylation following stimulation of peripheral blood cells with IFNα (Figure 4B). The relative expression levels for *Cxcl9*, *Ccl19* and *Epsti1*, were each decreased >10-fold in lacrimal glands of *Ifnar1*-deficient male NOD mice compared to wild-type male NOD mice and were comparable to relative expression levels in lacrimal glands of wild-type female NOD mice (Figure 4C). A decrease in relative expression of these genes in *Ifnar1*-deficient female NOD mice compared to wild-type female NOD mice was not statistically significant (Figure 4C). Relative expression levels for *Ubd* in lacrimal glands of *Ifnar1*-deficient male NOD mice were intermediate between those of wild-type male and wild-type female NOD mice but these differences were not statistically significant. These data suggested that type I IFN signaling contributed to the upregulated expression of several disease-relevant genes in lacrimal glands of wild-type NOD mice and that type I IFN signaling may be a key early signal in the development of dacryoadenitis. To address the role of type I IFN signaling in the development of dacryoadenitis, we quantified dacryoadenitis in wild-type and *Ifnar1*-deficient NOD mice. These analyses demonstrated a marked decrease in inflammation in lacrimal glands from *Ifnar1*-deficient male NOD mice compared to wild-type male NOD mice (Figure 4D). Little to no dacryoadenitis developed in either wild-type or *Ifnar1*-deficient female NOD mice (Figure 4D). Thus, type I IFN signaling contributed to the male-specific upregulation of disease-relevant genes (*Cxcl9*, *Ccl19*, *Epsti1*) and was required for dacryoadenitis development in male NOD mice.

## 3. Discussion

Sjögren syndrome is a complex autoimmune disease with little known of the earliest mechanisms driving the initial T-cell infiltration into the lacrimal or salivary glands. Diagnosis in humans may not occur until decades after the autoimmune response begins [1,2] making these initial infiltrating events difficult to study in humans. NOD mice spontaneously develop dacryoadenitis and sialadenitis similar to human Sjögren syndrome providing a model to study the early events required for disease initiation. One feature of NOD mice that has been poorly understood is the male-specific occurrence of dacryoadenitis. This has been demonstrated to be dependent on male sex hormones [7,8,10], though how androgens promote lacrimal gland infiltration by T cells is not well understood. We have previously demonstrated that male-specific dacryoadenitis in NOD mice is also associated with dysfunction of lacrimal gland-protective Tregs [8]. This Treg-dysfunction was lacrimal gland-specific as male NOD mice had Tregs capable of preventing salivary gland inflammation [6]. We considered the possibility that testosterone affects Treg function thus driving dacryoadenitis in NOD mice. However, in vivo depletion of Tregs in castrated mice failed to induce dacryoadenitis. Notably, Treg-depletion was comparable at 3 and 6 weeks following initial antibody treatment but by take-down at 10 weeks following initial treatment, the castrated mice had a higher proportion of Tregs within cervical lymph nodes compared to sham-castrated mice suggesting that the absence of testosterone may have provided a favorable environment for Tregs to begin to repopulate following withdrawal of Treg-depleting antibody treatment. While this may have contributed to the lack of disease development in the Treg-depleted, castrated mice, we would have expected that the significantly decreased Treg proportions in these mice compared to the isotype-treated, castrated mice would provoke some (even slight) increase in dacryoadenitis in the Treg-depleted group. However, this was not the case, suggesting that in addition to any effects on Tregs, testosterone must also be required to influence the effector side of the regulatory/effector imbalance required for autoimmunity. While this may be due to a direct effect on effector T cells, our investigations suggested the effect was on innate immune cells or epithelial cells within lacrimal glands.

We accessed prior microarray studies to examine testosterone effects on NOD and MRL/lpr mouse lacrimal glands in the context of autoimmune dacryoadenitis. We validated the expression of four genes that were upregulated in a sex- or testosterone-specific manner in the microarray studies in disease-related contexts including in male NOD mice compared to female NOD mice and in sham-castrated male NOD mice compared to castrated male NOD mice. Of these disease-relevant genes, expression of the chemokine genes *Cxcl9* and *Ccl19* were both slightly but significantly, upregulated in lacrimal glands of lymphocyte-deficient male NOD-SCID mice compared to female NOD-SCID mice and, thus, may be key signals directing early T-cell infiltration of the glands. However, the male-specific increased relative expression in lacrimal glands of NOD-SCID mice was not as robust as that in NOD mice, suggesting the possibility that the relative upregulation of these genes may be amplified by the inflammatory milieu. While upregulated by testosterone in NOD mice, these genes were found to be downregulated by testosterone in MRL/lpr mice but in both cases (NOD and MRL/lpr mice) these genes were upregulated in the context of lacrimal gland inflammation. In humans, Sjögren syndrome is more commonly diagnosed in females, so the promotion of lacrimal gland disease by testosterone in male NOD mice is of uncertain significance with respect to human disease. We study the NOD mouse model to understand how testosterone promotes lacrimal gland autoimmunity so that we may then do translational studies to determine if similar downstream mechanisms of T-cell infiltration of lacrimal glands are also apparent in human Sjögren syndrome. While testosterone itself may not be a required inciting factor for disease in humans, the downstream mechanisms of lacrimal gland autoimmunity may be comparable. As discussed below, several of our findings in this study, including upregulation of some of the genes identified here as well as the role of type I IFN, have also been identified in other models of Sjögren syndrome and implicated in human Sjögren syndrome suggesting the mechanisms driving immune dysregulation in NOD mice may reflect those in human Sjögren syndrome regardless of this sex and hormone discrepancy.

*Cxcl9* has been implicated in several contexts in Sjögren syndrome. *Cxcl9* was upregulated in salivary glands in several mouse models of Sjögren syndrome [20,22] and in lacrimal glands of the NFS/sld model of Sjögren syndrome [24]. Similar to our findings, *Cxcl9* expression was greatly increased in lacrimal glands of 20-week-old male NOD mice compared to females [30]. This upregulation was not altered in response to treatment with lymphotoxin-β receptor blockade, which was the focus of the study, so further evaluation was not done. In studies of another model of Sjögren syndrome, C57BL/6.NOD-*Aec1Aec2* mice, lacrimal gland gene expression changes over time were measured by microarray analyses [33,34]. *Cxcl9* was found to be modestly increased in lacrimal glands of 8 and 16-week-old C57BL/6.NOD-*Aec1Aec2* mice but was not increased at 12 or 20 weeks of age [33]. The upregulation in this study was relative to expression in lacrimal glands of 4-week-old C57BL/6.NOD-*Aec1Aec2* mice, which have normal glandular architecture compared to control strains. Since C57BL/6.NOD-*Aec1Aec2* mice are distinct from NOD and MRL/lpr mice, it is possible that the contributions of *C*XCL9 to lacrimal gland disease is different in this particular mouse strain. Another possibility is that some level of *Cxcl9* upregulation is already occurring in lacrimal glands of these mice at 4 weeks of age, which would not have been detected and would have blunted the measures of upregulation in lacrimal glands at later time points as they used the 4-week gene expression levels as the baseline to which all others were compared. Our finding of slight but significant upregulation of *Cxcl9* in lacrimal glands of NOD-SCID mice suggest that the events leading to *Cxcl9* gene upregulation in lacrimal glands precedes lymphocytic infiltration, so it is possible that it may already be upregulated at an early time point even without significant gland architectural changes evident on histology. In C57BL/6 mice, *Cxcl9* expression in salivary glands was upregulated following administration of the TLR3 ligand, poly I:C and was associated with type I and type II IFN production, IL7 upregulation and NK cells [35]. Treatment of female NOD mice with IL7Rα-blocking antibodies decreased *Cxcl9* expression in salivary glands and also decreased expression of the genes for the two other CXCR3 ligands *Cxcl10* and *Cxcl11*, all of which were associated with decreased T-cell infiltration into the salivary glands [23]. In human studies, salivary gland epithelial cells isolated from healthy individuals and treated with anti-SSA/Ro antibodies isolated from Sjögren syndrome patients showed an increase in the expression of *Cxcl9* among genes encoding other inflammatory cytokines and chemokines [25]. CXCL9 protein levels (along with CXCL10 and CXCL11) were elevated in tears and conjunctival epithelium from Sjögren syndrome patients compared to controls samples [27]. *Cxcl9* gene expression levels were also higher in conjunctival epithelium from Sjögren syndrome patients compared to controls [26]. To our knowledge no studies of *Cxcl9* expression in lacrimal glands of Sjögren syndrome patients have been reported.

*Ccl19* has also been implicated in Sjögren syndrome in several studies, though much less has been reported compared to that for *Cxcl9*. Similar to *Cxcl9*, *Ccl19* gene expression was upregulated in salivary glands of a NOD-related mouse model of Sjögren syndrome [20]. *Ccl19* was also similarly upregulated in lacrimal glands of male compared to female NOD mice [30]. While expression of *Cxcl9* was not altered with lymphotoxin-β receptor blockade, treatment of male NOD mice with lymphotoxin-β receptor blockade resulted in a marked downregulation of *Ccl19* suggesting that expression of *Cxcl9* and *Ccl19* may be regulated differently [30]. Increased expression of *Ccl19* was detected in lacrimal glands of C57BL/6.NOD-*Aec1Aec2* mice early in disease development, specifically with peak expression at 8 weeks followed by a steady decrease in degree of upregulation (but still upregulated >5-fold compared to expression in lacrimal glands of 4-week-old C57BL/6.NOD-*Aec1Aec2* mice) through 20 weeks [33]. CCL19 plays a role in trafficking of lymphocytes to secondary lymphoid organs as well as to tertiary lymphoid structures such as those that occur in target organs in the context of several autoimmune diseases. However, whether these tertiary lymphoid structures develop in lacrimal glands in Sjögren syndrome has not been reported and was not evaluated here.

Of the other disease-relevant genes identified in our study, *Epsti1* was upregulated in patients with Sjögren syndrome [28,29]. *Epsti1* upregulation was also noted in systemic lupus erythematosus [36] and rheumatoid arthritis patients [37]. In the latter study, *Epsti1* was included in a set of genes used to calculate an IFN score, which they found correlated with therapeutic response to treatment with the B cell-depleting therapy rituximab. While the role of *Epsti1* in the early inflammation of lacrimal glands is not yet known, a recent study identified a role for *Epsti1* in modulating polarization of macrophages to the inflammatory M1 type with *Epsti1*-deficient macrophages demonstrating a blunted upregulation of M1-type genes (including *Cxcl9*) following exposure to inflammatory stimuli [38]. *Ubd* (also known as *Fat10*) is expressed by cells of the immune system at steady-state but may be upregulated by nearly any cell type in the context of inflammation [39,40,41,42,43]. Interestingly, *Ubd* was shown to modulate pattern recognition receptors in the context of an anti-viral inflammatory response [44], which may contribute to the early innate immune response leading to the attraction of T cells to the lacrimal glands. Of note, studies utilizing microarray analyses of lacrimal glands from C57BL/6.NOD-*Aec1Aec2* mice, which also develop lacrimal gland autoimmunity, did not report upregulation of *Epsti1* or *Ubd* early in the course of disease [33,34]. These studies specifically characterized temporal changes in differential gene expression in C57BL/6.NOD-*Aec1Aec2* mice over time compared to that of 4-week-old C57BL/6.NOD-*Aec1Aec2* mice. If these genes were upregulated at this early time point without further upregulation in the context of disease development, then they would not be detected as differentially expressed in those studies. However, our study did not identify upregulation of these two genes in the absence of lymphocytic infiltration (e.g., in lacrimal glands of NOD-SCID mice), so they may not be upregulated at early time points prior to significant T-cell infiltration. Since C57BL/6.NOD-*Aec1Aec2* mice contain mostly C57BL/6 DNA (with two large stretches of NOD DNA on chromosomes 1 and 3), it is entirely possible that they may have some differences in disease pathogenesis compared to NOD mice. On the other hand, the detection of the genes identified in our study were based on microarray data from two distinct mouse strains (NOD and MRL/lpr) making them seemingly more likely to be relevant in multiple models of disease. Thus, further studies to reconcile these differences in lacrimal gland autoimmunity in these different mouse strains are warranted.

One common feature among the disease-relevant genes upregulated in a male-specific manner was their upregulation in response to type I and type II IFN. Since NOD mice deficient in type II IFN or type II IFN signaling were not protected from development of dacryoadenitis [32], we focused on type I IFN. We found that upregulation of *Cxcl9*, *Ccl19* and *Epsti1* was altered in NOD mice deficient in type I IFN signaling and these mice also failed to develop dacryoadenitis. In a related mouse strain, the C57BL/6.NOD-*Aec1Aec2* mouse, disruption of type I IFN signaling resulted in decreased sialadenitis and dacryoadenitis [11] but a role for type I IFN in dacryoadenitis in NOD mice has been questioned [3]. Here, we report a requisite role for type I IFN signaling in male-specific dacryoadenitis in the NOD mouse model of Sjögren syndrome.

Together, these data suggest a model in which androgen exposure affects cells within the lacrimal glands of NOD mice leading to an innate immune response (including type I IFN production and subsequent upregulation of *Cxcl9*, *Ccl19* and *Epsti1*) that triggers the adaptive immune response (i.e., T-cell infiltration into lacrimal glands). Lacrimal gland dysregulation has been described in young male NOD mice including disorganized extracellular matrix, disruption of normal exocytic pathways of acinar cells, altered lipid homeostasis and other cytologic changes of acinar cells on an ultrastructural level [45,46,47,48]. The cause of these morphological and cellular changes is still not clear but our findings indicate that testosterone-mediated type I IFN signaling may be a likely triggering factor.

## 4. Materials and Methods

### 4.1. Mice

Male and female NOD mice (NOD/ShiLtJ), castrated and sham-castrated NOD mice and NOD-SCID mice (NOD.CB17-*Prkdc^scid^*/J) were purchased from The Jackson Laboratory (Bar Harbor, ME, USA). *Ifnar1*-mutant NOD mice were generated by the Australian Phenomics Facility (Monash University, Clayton, Australia) and maintained at St Vincent’s Institute (Fitzroy, Australia). Briefly, CRISPR/Cas9 mutagenesis was used to target *Ifnar1* using sgRNA 5′-GCTCGCTGTCGTGGGCGCGG-3′, which targets the first exon of *Ifnar1*. Resulting G_0_ offspring generated from embryos injected with Cas9 mRNA and the sgRNA were screened by sequencing PCR-derived amplicons for the targeted region in *Ifnar1*. Mutant allele *em16* was identified as a 5 bp deletion that causes a frame-shift mutation and a premature stop-codon. This G_0_ founder mouse was used to establish an *Ifnar1*-mutant NOD mouse line (NOD.*Ifnar1^em16^*^/*em16*^). Mice are genotyped by mutation-specific Taqman probe assays (LightCycler 480 Probes Master Mix, Roche Diagnostics, Indianapolis, IN, USA) using a LightCycler 480II (Roche Diagnostics) using standard conditions and the following *Ifnar1*-specific primers and fluorescently labelled probes (5’–3’): Forward primer CGGCCTCCCAAGACGAT, Reverse Primer CTGCAGCTGAGGGTAGCA, Wildtype probe CGCCGCGCCCACGA, Mutation probe CAGGGCCGCCCACGA. All mice were monitored for the presence of glucosuria using Diastix urine dipsticks (Bayer Diagnostics, Whippany, NJ, USA). A positive test was indicative of autoimmune diabetes development; however, no mice in these studies tested positive for diabetes. Mice were maintained and used in accordance with the University of Iowa Animal Care and Use Committee Guidelines (#6021655, 10 May 2016) or as approved by the Animal Ethics Committee at St Vincent’s Institute (#AEC 028/16, 4 November 2015).

### 4.2. In Vivo Treg Depletion

Male NOD mice were castrated or sham-castrated at 4–5 weeks old. Tregs were depleted in vivo by treating mice weekly for 4 doses (0.5 mg/dose given intraperitoneally) of anti-CD25 monoclonal antibody (clone PC-61.5.3) or rat IgG1 isotype control antibody (clone HRPN) (Bio X Cell, West Lebanon, NH, USA). Treatment with Treg-depleting or control antibody was initiated 1 week after castration (age 5–6 weeks) and mice were euthanized 10 weeks after first antibody treatment for histological and flow cytometric analyses.

### 4.3. Histological Characterization of Lacrimal Gland Inflammation

Quantification of lacrimal gland inflammation by focus scoring was performed as previously described [49]. Briefly, hematoxylin and eosin (H&E)-stained sections of formalin-fixed, paraffin-embedded, exorbital lacrimal glands were analyzed by standard light microscopy using a 10× objective. Foci composed of a minimum of 50 mononuclear cells were counted and slides scanned to obtain digital images using PathScan Enabler IV (Meyer Instruments, Houston, TX, USA). Tissue areas were measured using ImageJ software [50] and focus scores quantified as number of inflammatory cell foci per 4 mm^2^ tissue area. Representative low-magnification digital images were obtained with a PathScan Enabler IV slide scanner (Meyer Instruments).

### 4.4. Lymphocyte Isolation and Flow Cytometry

Cervical lymph nodes were dissociated with the end of a plunger of a 3 mL syringe through a 70 µm nylon mesh in RPMI (Life Technologies, Waltham, MA, USA) supplemented with 10% fetal bovine serum, 100 U/mL penicillin, 100 µg/mL streptomycin and 50 µM β-mercaptoethanol (complete RPMI). Red blood cells were removed through treatment with ACK lysis buffer (Lonza, Mapleton, IL, USA). Single cell suspensions were stained with fluorophore-conjugated monoclonal antibodies: CD3ε (145-2c11), CD4 (GK1.5 or RM4-5), CD19 (1D3), CD25 (7D4), Foxp3 (FJK-16s) purchased from eBioscience (San Diego, CA, USA), BioLegend (San Diego, CA, USA), or BD Biosciences (San Jose, CA, USA). Intracellular staining for Foxp3 was performed per manufacturer’s protocol (eBioscience). Peripheral blood mononuclear cells were collected and isolated with Histopaque-1083 (Sigma-Aldrich) as previously described [49]. Phosphorylated STAT1 (pSTAT1) staining, to confirm disruption of IFNAR1 expression, was performed on whole blood stimulated with recombinant mouse IFNα (1000 U/mL, PBL Assay Science, Piscataway, NJ, USA) or recombinant mouse IFNγ (100 U/mL, BioLegend) for 30 min at 37 °C. Red blood cells were lysed (155 mM NH_4_CL, 10 mM Tris-HCl, pH 7.5). Remaining leukocytes were washed with PBS, then fixed and permeabilized with methanol and stained with anti-CD4 (clone GK1.5), anti-CD8 (clone 53-6.7) and anti-pSTAT1 (clone 4A, BD Biosciences) monoclonal antibodies. Flow cytometry data acquisition was performed on a BD LSR II, BD LSR Fortessa, or BD Accuri C6 and subsequently analyzed with FlowJo software (Treestar Inc., Ashland, OR, USA).

### 4.5. Analysis of Publicly Available Microarray Data

Microarray data sets comparing lacrimal gland gene expression in NOD mice and MRL/lpr mice were downloaded through the National Center for Biotechnology Information’s Gene Expression Omnibus via series accession number GSE5876 (male versus female) and GSE5877 (testosterone versus placebo treated females). They were analyzed with GeneSifter software (Geospiza, Seattle, WA, USA) to identify genes significantly upregulated at least two-fold in a lacrimal gland disease-associated context (i.e., male NOD mice greater than female NOD mice, testosterone-treated female NOD mice greater than placebo-treated female NOD mice; female MRL/lpr mice greater than male MRL/lpr mice, placebo-treated female MRL/lpr mice greater than testosterone-treated MRL/lpr mice). Details of these studies are described elsewhere [18,19]. Gene identifiers for those genes upregulated in each disease-relevant comparison were analyzed with Venny 2.1.0 (bioinfogp.cnb.csic.es/tools/venny/) [51] to identify the genes within the intersections of all groups.

### 4.6. Quantitative RT-PCR

Mouse lacrimal glands were removed and placed in RNA*later* (Invitrogen, Waltham, MA, USA). Tissue samples were lysed and homogenized using the lysis buffer provided in the RNeasy Plus Mini Kit (Qiagen, Valencia, CA, USA) using a disposable micro homogenizer. Total RNA extraction was performed using the RNeasy Plus Mini Kit according to manufacturer’s instructions. The SuperScript II Reverse Transcriptase Kit (Invitrogen) and random primers (Invitrogen) were used to make cDNA. PCR was performed in the ABI 7500 or ABI 7900 HT Real Time PCR Systems (Applied Biosystems, Foster City, CA, USA) using the Power SYBR Green PCR Master Mix (Applied Biosystems) as described [52]. The copy number (number of transcripts) of amplified product was calculated from a standard curve obtained by plotting known input concentrations of plasmid DNA. Expression levels of each gene were normalized to *Gapdh*. Primer sequences are listed (Table 4).

### 4.7. Statistical Analysis

Statistical analyses were performed with Prism 7.00 (GraphPad, San Diego, CA, USA). Mann-Whitney test was used for two-group comparisons of non-normally distributed data (focus scores, gene expression). All two group comparisons were two-tailed. One way ANOVA with Holm-Sidak multiple comparisons post-test was used for multi-group analyses of data that approximated normal distribution (flow cytometry data). Kruskal-Wallis with Dunn multiple comparisons post-test was used for multi-group comparisons of non-normally distributed data (focus scores, gene expression). For gene expression studies, ratios were log-transformed prior to statistical analyses. Repeated measures two-way ANOVA was used for multiple group comparisons over time (peripheral blood mononuclear cell flow cytometry data) with individual comparisons at each time point corrected for multiple comparisons by Tukey post-test. *p* values < 0.05 were considered significant.

## Figures and Tables

**Table 1:** Genes upregulated in the context of lacrimal gland disease in NOD and MRL/lpr mice.

Gene ID	Gene Identifier	Gene Name	Ratios ^1^
*Cxcl9*	NM_008599	Chemokine (C-X-C motif) ligand 9	7.65 (2.53–28.95)
*Ubd*	NM_023137	Ubiquitin D	7.75 (3.01–20.37)
*Ifi47*	NM_008330	Interferon γ inducible protein 47	4.40 (2.75–8.2)
*Tgtp*	NM_011579	T-cell specific GTPase	5 (3.01–7.56)
*Epsti1*	AK017174	Epithelial stromal interaction 1	4.1 (2.6–6.79)
*Ccl19*	NM_011888	Chemokine (C-C motif) ligand 19	3.37 (2.05–5.51)
*Clec7a*	NM_020008	C-type lectin domain family 7, member a	3.13 (2.9–5)

^1^ Ratios are listed as median (interquartile range) and include the range of ratios of mean levels of gene expression for each of the comparisons depicted in Figure 2 with numerators representing the group developing dacryoadenitis and denominators representing the group protected from dacryoadenitis. Specifically, these ratios include male NOD/female NOD, testosterone-treated female NOD/placebo-treated female NOD, female MRL/male MRL, placebo-treated female MRL/testosterone-treated female MRL. The ratios were generated by GeneSifter analyses for each of the two-group comparisons from the Affymetrix GeneChip Mouse Expression Array 430A and CodeLink UniSet Mouse 20K I Bioarray platforms.

**Table 2:** Validation of genes upregulated in the context of lacrimal gland disease by qPCR.

Gene ID	Relative Expression ^1^		*p* Value ^2^	Ratio ^3^
	Male NOD	Female NOD		
*Cxcl9*	0.419 (0.352–0.895)	0.013 (0.003–0.016)	0.006	32.2
*Ubd*	0.024 (0.023–0.027)	0.0004 (0.0001–0.002)	0.001	60
*Epsti1*	0.134 (0.075–0.172)	0.029 (0.007–0.038)	0.0003	4.6
*Ccl19*	0.099 (0.078–0.193)	0.0063 (0.0057–0.023)	0.001	15.7
*Clec7a*	0.059 (0.029–0.156)	0.016 (0.01–0.208)	0.463	3.7

^1^ RNA was isolated from lacrimal glands of male and female NOD mice. Gene expression was quantified using a standard curve and normalized to *Gapdh*. Numbers are presented as median (interquartile range). ^2^ *p* Values by Mann-Whitney test applied to log-transformed ratios of each gene normalized to *Gapdh.* ^3^ Ratios of median values of relative expression (male divided by female).

**Table 3:** Downregulation of disease-relevant genes in lacrimal glands of castrated male NOD mice.

Gene ID	Relative Expression ^1^		*p* Value ^2^	Ratio ^3^
	Sham-Castrated	Castrated		
*Cxcl9*	0.520 (0.407–0.589)	0.051 (0.035–0.071)	<0.01	10.2
*Ubd*	0.021 (0.019–0.030)	0.0012 (0.001–0.0017)	<0.01	17.5
*Epsti1*	0.079 (0.061–0.108)	0.028 (0.024–0.031)	<0.01	2.8
*Ccl19*	0.107 (0.088–0.114)	0.017 (0.015–0.024)	<0.01	6.3
*Clec7a*	0.082 (0.081–0.1)	0.038 (0.031–0.044)	<0.01	2.2

^1^ RNA was isolated from lacrimal glands of sham-castrated and castrated male NOD mice. Gene expression was quantified by qPCR using a standard curve and normalized to *Gapdh*. Numbers are presented as median (interquartile range). ^2^ *p* Values by Mann-Whitney test applied to log-transformed ratios of each gene normalized to *Gapdh.* ^3^ Ratios of median values of relative expression (sham-castrated divided by castrated).

**Table 4:** Primer sequences for quantitative RT-PCR.

Gene ID	Forward (5′–3′)	Reverse (5′–3′)
*Cxcl9*	CCGAGGCACGATCCACTACA	CGAGTCCGGATCTAGGCAGGT
*Ubd*	ACCAGATCCTTCTGCTAGACT	GGGTAAGGTGGATAGTGGTTTC
*Epsti1*	GAGAGCAGAAGGCAGAAGATAC	CGTTGTGGCACCAAGTAGA
*Ccl19*	ATGTGAATCACTCTGGCCCAGGAA	AAGCGGCTTTATTGGAAGCTCTGC
*Clec7a*	ATCAGCATTCTTCCCCAACTCG	CAGTTCCTTCTCACAGATACTGTATGA
*Gapdh*	TTCACCACCATGGAGAAGGC	GGCATGGACTGTGGTCATGA

## Abbreviations

H&E	Hematoxylin and eosin
IFN	Interferon
MRL/lpr	Murphy Roths Large/lymphoproliferation
NOD	Nonobese diabetic
SCID	Severe combined immunodeficiency
Tregs	Regulatory T cells
